# The Liquid Young’s Law on SLIPS: Liquid–Liquid Interfacial Tensions and Zisman Plots

**DOI:** 10.1021/acs.langmuir.2c01470

**Published:** 2022-08-03

**Authors:** Glen McHale, Nasser Afify, Steven Armstrong, Gary G. Wells, Rodrigo Ledesma-Aguilar

**Affiliations:** Institute for Multiscale Thermofluids, School of Engineering, The University of Edinburgh, Edinburgh EH9 3FB, United Kingdom

## Abstract

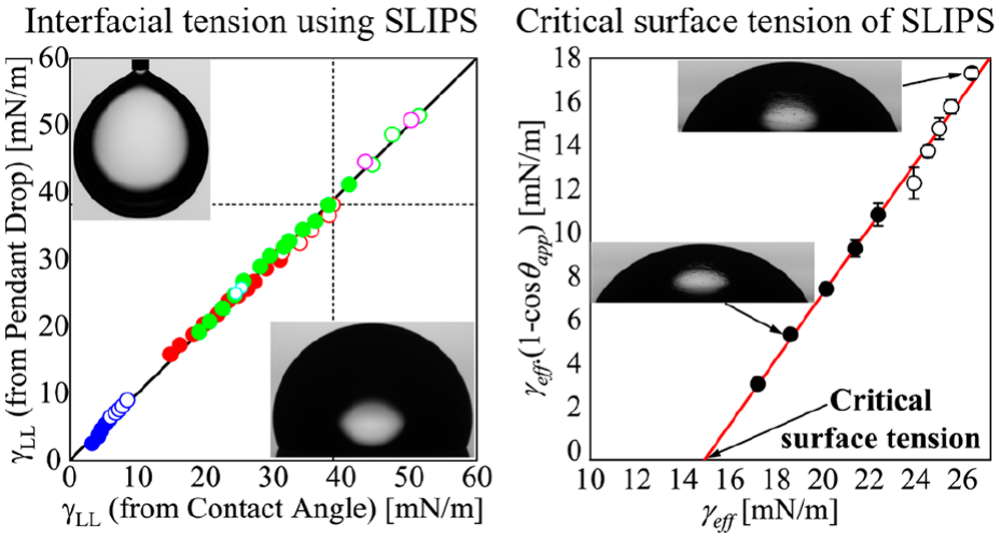

Slippery liquid-infused porous surfaces (SLIPS) are an innovation that reduces droplet-solid contact line pinning and interfacial friction. Recently, it has been shown that a liquid analogue of Young’s law can be deduced for the apparent contact angle of a sessile droplet on SLIPS despite there never being contact by the droplet with the underlying solid. Since contact angles on solids are used to characterize solid–liquid interfacial interactions and the wetting of a solid by a liquid, it is our hypothesis that liquid–liquid interactions and the wetting of a liquid surface by a liquid can be characterized by apparent contact angles on SLIPS. Here, we first present a theory for deducing liquid–liquid interfacial tensions from apparent contact angles. This theory is valid irrespective of whether or not a film of the infusing liquid cloaks the droplet–vapor interface. We show experimentally that liquid–liquid interfacial tensions deduced from apparent contact angles of droplets on SLIPS are in excellent agreement with values from the traditional pendant drop technique. We then consider whether the Zisman method for characterizing the wettability of a solid surface can be applied to liquid surfaces created using SLIPS. We report apparent contact angles for a homologous series of alkanes on Krytox-infused SLIPS and for water–IPA mixtures on both the Krytox-infused SLIPS and a silicone oil-infused SLIPS. The alkanes on the Krytox-infused SLIPS follow a linear relationship in the liquid form of the Zisman plot provided that the effective droplet–vapor interfacial tension is used. All three systems follow a linear relationship on a modified Zisman plot. We interpret these results using the concept of the critical surface tension (CST) for the wettability of a solid surface introduced by Zisman. In our liquid surface case, the obtained critical surface tensions were found to be lower than the infusing liquid–vapor surface tensions.

## Introduction

The concept of an equilibrium contact angle, θ_e_, and its mathematical relationship to the solid–vapor (γ_SV_), solid–liquid (γ_SL_), and liquid–vapor (γ_LV_) interfacial tensions through Young’s law is fundamental to the concepts of the wettability of a solid by a liquid.^1,2^ However, solid surfaces tend not to be homogeneous, but have roughness and chemical heterogeneity which causes contact line pinning. Surfaces are then characterized by a maximum and a minimum contact angle referred to as the advancing, θ_A_, and receding, θ_R_, contact angles between which the equilibrium contact angle is assumed to exist. The angular range, Δθ = θ_A_ – θ_R_, defines the contact angle hysteresis (CAH), and for many solids, this can encompass a considerable numerical range of many tens of degrees.^3^ Despite this limitation, the measured (static) contact angle, θ_s_, is often assumed to provide an approximation to θ_e_ and knowledge of the wettability of the solid surface. Thus, for example, one may refer to a hydrophilic, hydrophobic, or superhydrophobic surface as defined by θ_e_ < 90°, θ_e_ > 90°, or θ_e_ > 150°, the ability of a liquid (L) to spread across and form a film on a flat smooth solid surface (S) in the presence of another fluid (V) through expressing the spreading coefficient, *S*_LS(V)_ = γ_SV_ – (γ_SL_ + γ_LV_), in terms of the contact angle using *S*_LS(V)_ = γ_LV_(cos θ_e_ – 1) or the work of adhesion, *W*_LS(V)_ = γ_SV_ + γ_LV_ – γ_SL_, via the Young–Dupré equation *W*_LS(V)_ = γ_LV_(1 + cos θ_e_).^2,4,5^ Contact line pinning is also the basis of the concept of static droplet friction with the pinning force given by *F*_p_ = *kw*γ_LV_(cos θ_R_ – cos θ_A_), where *w* is the droplet contact width, and *k* is a numerical constant.^6−10^ The explicit dependence of this equation on CAH can be seen by the alternative formulation *F*_p_ = *kw*γ_LV_ sin θ_e_Δθ, which is a droplet form of Amontons’ laws of solid-on-solid friction with a static coefficient of friction, μ_s_ = *k*Δθ/π, relating the pinning force to a normal component of the capillary force, *F*_N_ = π*w*γ_LV_ sin θ_e_.^11−13^

Motivated by a desire to create robust synthetic slippery surfaces (defined as low-contact-angle hysteresis surfaces with typically Δθ < 2.5°) capable of shedding liquids and, in contrast to superhydrophobic surfaces, pressure-stable, Wong et al. introduced the concept of slippery liquid-infused porous surfaces (SLIPS)^14^ (for recent reviews, see refs (13 and 15−17)). These surfaces use an infusing liquid (also referred to here as a lubricant) which completely and stably, preferentially wets the solid and acts as an immiscible lubricant for another contacting liquid. SLIPS are one possible state of lubricant impregnated surfaces (LISs), which arise in the coating of textured solids and the study of hemiwicking.^18−20^ A small sessile droplet on a SLIP surface is a spherical cap with a circular arc side profile from its apex until the profile approaches the liquid-infused solid surface where a wetting ridge occurs. The size of the wetting ridge is related to the existence of the infused-liquid and depends on the excess amount of lubricant on the SLIPS.^21−24^ A further complication is that the infused-liquid can spread across the droplet–vapor interface and cloak the droplet if the spreading coefficient *S*_L_i_L_d_(V)_ = γ_L_d_V_ – (γ_L_d_L_i__ + γ_L_i_V_) ≥ 0 where γ_*IJ*_ are the various fluid–fluid interfacial tensions.^20,25^ In this paper, we use “L_i_” to indicate the infused-liquid (typically, but not necessarily, an oil), “L_d_” to indicate the liquid in the droplet, and “V” to indicate the surrounding vapor (in general, this could be a third immiscible liquid). We also limit our consideration to macroscopic quantities and modeling on the basis of interfacial tensions. Once the coating thickness approaches the range of forces between the molecules in a fluid, the energetics of a configuration of different fluids can no longer be modeled solely on the basis of interfacial tensions.^13,26^

Despite the complication of droplet cloaking, providing an effective surface tension γ_eff_ for the droplet–vapor interface is introduced, a liquid analogue of Young’s law for the apparent contact angle can be defined in the limit of infinitesimally thin lubricant layers^23^ (see also refs (27 and 28)), i.e.
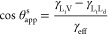
1where
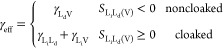
2For thicker lubricant layers, the apparent contact angle is defined as the tangent angle at the inflection point in the side profile of a droplet.^21^ As the lubricant becomes thicker, the excess lubricant causes the apparent contact angle to decrease by rotation of the Neumann triangle at the inflection point of the side profile.^23,29^ On LIS surfaces where the lubricant does not form a continuous layer across the top of the underlying solid, the apparent contact angle is given by a Cassie-weighted average of the Young’s law for the solid and the liquid Young’s law,^13^ i.e.
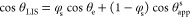
3where
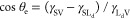
4is Young’s law on the solid surface,^2^ and φ_s_ and (1 – φ_s_) are the solid and infused-liquid fractions of the surface, respectively. Equation 3 is only valid when the interfacial tensions are such that the LIS surface is energetically stable in air and on immersion in the droplet liquid. A simple, but profound, observation is that the liquid analogue of Young’s law arises from the replacement of the symbol “S” in eq 4 by “L_i_” to give eq 3; i.e., solid is replaced by infused-liquid. Thus, while contact angles on a solid surface provide information about the solid–liquid interfacial tension γ_SL_, on a thin infused-liquid surface they provide information about the liquid–liquid interaction γ_L_i_L_d__. Moreover, the concept of wettability of a solid surface by a liquid can be extended to the wettability of a thin liquid surface by another (immiscible) liquid.

In the remainder of this paper we first consider the theory of the liquid Young’s law and show how it can be applied to estimate liquid–liquid interfacial tensions. Knowledge of interfacial tension between liquids is important for assessing the quality of industrial products such as coatings, paintings, ink printing, detergents, cosmetics, pharmaceuticals, lubricants, pesticides, food products, and agrochemicals^30−32^ and is relevant to industrial production processes, including catalysis, adsorption, and distillation,^30^ and the monitoring of the quality of atmosphere^33^ and wastewater.^34^ We then provide a comparison of liquid–liquid interfacial tensions deduced using the liquid Young’s law to those deduced from the traditional pendant drop technique.^35,36^ We therefore provide a new technique complementing existing liquid–liquid interfacial tension measurement methods, such as the Du Nouy ring,^37^ Wilhelmy plate,^38^ rod,^39^ bubble pressure,^40^ drop volume, and pendant drop^41^ (for a review of methods, see ref (42)). Finally, we focus on extending the Zisman method^43−45^ for assessing the wettability of solid surfaces to the case of liquid surfaces by considering apparent contact angles of a homologous series of alkanes (formula C_*n*_H_2*n*+2_) and a series of water–isopropylalcohol (IPA) solutions on Krytox oil- and silicone oil-infused SLIP surfaces. We develop and discuss Zisman’s concept of a critical surface tension (CST), γ_C_, for the wetting of a solid surface in the context of a liquid-infused surface and investigate whether, in the liquid surface case, it tends to or is less than the infused-liquid–vapor interfacial tension, i.e., whether γ_C_ ≤ γ_L_i_V_ is valid.

## Theory of Liquid–Liquid Interfacial Tension from the Liquid Young’s Law

We assume a substrate, S, which has been infused by a completely wetting liquid which forms a stable and continuous thin film of the infused-liquid, L_i_. We consider small sessile droplets of a second immiscible liquid, L_d_, resting on this layer of infused-liquid without displacing it. This implies choices of solid and liquids such that the film of infused-liquid is stable to both the vapor and the droplet liquid, i.e.
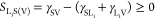
5and
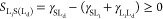
6It also implies that wetting of the solid by the infused-liquid is energetically preferred, i.e.
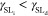
7

Provided these conditions are satisfied, we can rearrange eqs 1 and 2 to predict the liquid–liquid interfacial tension between the infused-liquid and the droplet liquid for “noncloaked” and “cloaked” droplets:
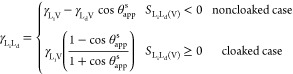
8Knowing the values of liquid–vapor interfacial tensions for the two liquids and measuring the apparent contact angle then gives two possible values for the liquid–liquid interfacial tensions depending on whether the droplet–vapor interface becomes cloaked or not. To choose between these two possible cases we make use of the spreading coefficient for the infused-liquid on the droplet liquid:
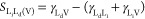
9We therefore select the case in eq 8 for γ_L_d_L_i__ which, when substituted into eq 9, is consistent with predicting *S*_L_i_L_d_(V)_ < 0 for noncloaked droplets or *S*_L_i_*L*_*d*(V)__ ≥ 0 for cloaked droplets.

## Experimental Methods

### Preparation of SLIPS

New microscope glass slides (25 mm × 75 mm) were cleaned by sonication, once for 15 min in a solution of deionized water (resistivity higher than 18 MΩ cm) and 2% v/v Decon 90 surfactant (Fisher Scientific). The samples were then sonicated twice in pure deionized water for 15 min and rinsed in fresh deionized water. The clean samples were dried in a fume hood for several hours and then coated with hydrophobized nanoparticles (Glaco Mirror Coat Zero, SOFT 99 Corp.) to obtain superhydrophobic surfaces. Glaco is an isopropyl alcohol suspension containing silica nanoparticles with a surface that is hydrophobically modified with a fluorosilane chemistry.^46,47^

Two coating methods were evaluated, namely, dip-coating and spray-coating. Each substrate was coated with 5 Glaco layers, with a drying time of 1 h in a fume hood between each two layers.^48^ Extensive contact angle characterization with water droplets showed that the dip-coated and spray-coated surfaces possess very similar superhydrophobic properties, with average static contact angles of 166.8° ± 2.0° and 164.4° ± 2.5°, respectively. The average contact angles after infusing the silicone and Krytox lubricating oils were 109.0° ± 0.2° and 119.6° ± 0.3° for the dip-coated samples and 109.1° ± 0.1° and 119.7° ± 0.2° for the spray-coated samples. These SLIPS showed very small contact angle hysteresis of 0.8° ± 0.3° in the case of silicone oil and 0.8° ± 0.2° in the case of Krytox oil.

Initial testing of the Glaco-based SLIP surfaces showed that alkane droplets displaced FC-70, Krytox, and silicone lubricating oils, and so we also prepared Teflon-based SLIP samples which were stable against droplets of the different alkanes. These choices are guided by knowledge of the critical surface tensions of smooth polymeric surface and surfaces composed of various end groups, and the surface tension of various liquids.^49^ This second set of Teflon-based hydrophobic surface glass substrates were prepared using Teflon AF1600. The solution was prepared by dissolving 0.5 wt % poly[4,5-difluoro-2,2-bis(trifluoromethyl)-1,3-dioxole-*co*-tetrafluoroethylene] in octadecafluorodecahydronaphthalene solvent. The mixture was left overnight under magnetic stirring at 60 °C. To enhance spreading on glass substrates, clean glass slides were further cleaned in a Henniker HPT-200 plasma cleaner for 10 min at 200 W power. A 300 mL solution was then spread on each glass substrate using another clean glass slide. The Teflon-coated slides were then dried on a hot plate at 155 °C for 1 h. The average static contact angle of water droplets on these hydrophobic surfaces was 123.2° ± 1.1°. The average contact angle after infusing the Krytox lubricating oils was 120.7° ± 0.8° for these Teflon-based samples.

To infuse lubricant into the various surfaces and create SLIPS, each substrate was dip-coated in a lubricant using a withdrawal speed of 0.1 mm/s.^18^ Each sample was then rinsed with deionized water, until no wetting ridges were observed during contact angle measurements. For any pair of the droplet and infused-liquids, a fresh sample was used to avoid effects of lubricant degradation and/or contamination. Infusing liquids included silicone oil (20 cSt at 25 °C, Sigma-Aldrich), Krytox vacuum oil 1506 (62 cSt at 20 °C, Sigma-Aldrich), and Fluorinert FC-70 (11.0–17.0 cSt at 25 °C, Sigma-Aldrich). Edible infused-liquids included olive and avocado oils (Sainsbury’s UK) and C8-MCT oil (Wellgard). More details on SLIPS preparation can be found elsewhere.^28,50^

For droplets, two series of liquids were used. The first series of liquids was a set of homologous alkanes (purity ≥99%, Sigma-Aldrich) including pentane (C_5_H_12_), hexane (C_6_H_14_), heptane (C_7_H_16_), octane (C_8_H_18_), nonane (C_9_H_20_), decane (C_10_H_22_), undecane (C_11_H_24_), dodecane (C_12_H_26_), tridecane (C_13_H_28_), and hexadecane (C_16_H_34_). The second series consisted of several isopropanol (purity 99.8%, Fisher Scientific) deionized water solutions, with Isopropanol concentration ranging from 0 to 40% v/v.

### Contact Angle Measurements

**Figure 1:**
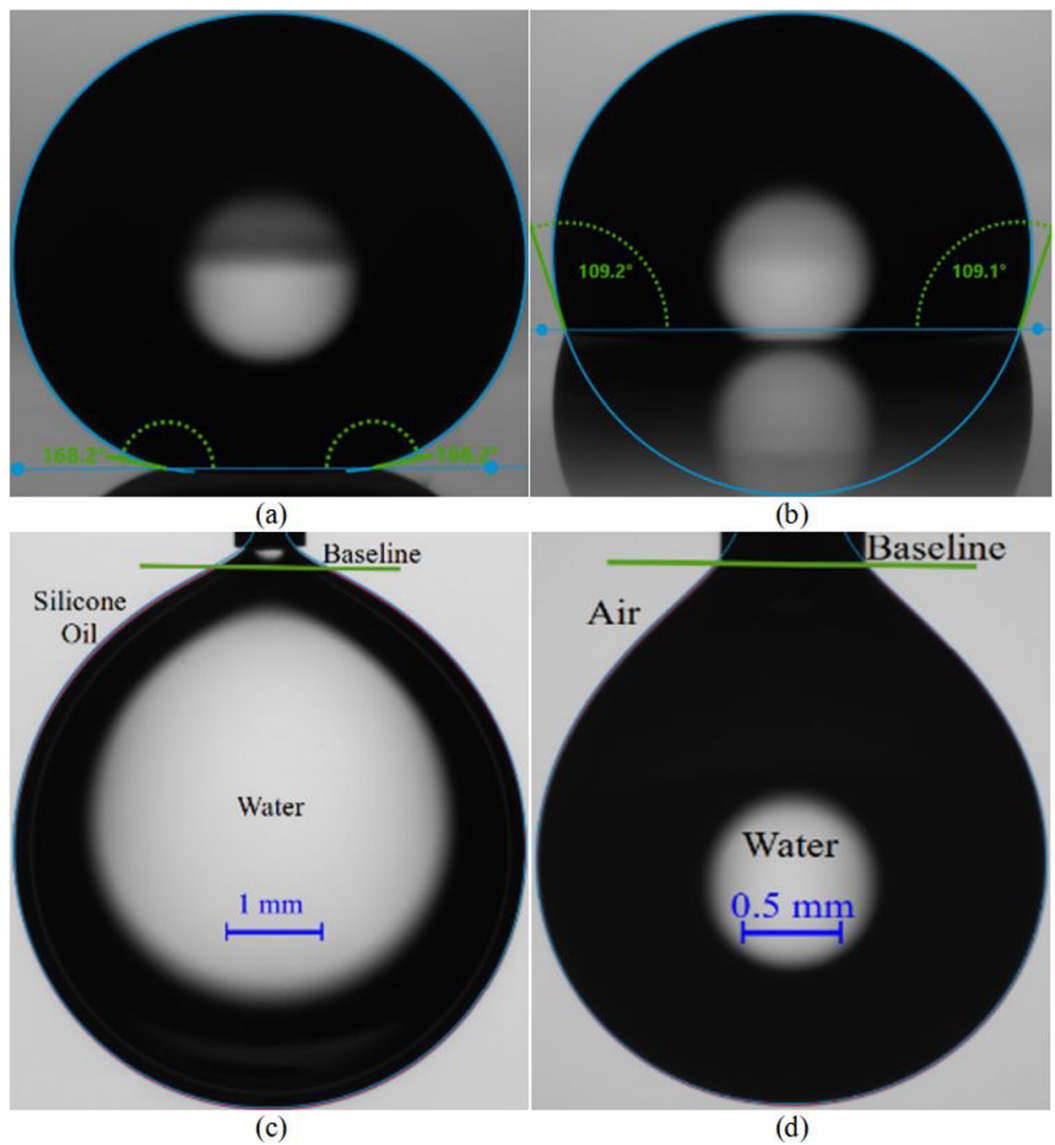
Sample images of contact angle and pendant drop interfacial tension measurements: (a) Water droplet setting on Glaco superhydrophobic surface. (b) Water droplet setting on a SLIPS employing silicone oil as a lubricant. (c) Water pendant drop in air. (d) Water pendant drop in silicone oil inside glass cuvette.

Apparent contact angles θ_app_ were measured using a Krüss droplet shape analyzer (DSA25S) at room temperature (17–25 °C). Unless, otherwise mentioned, the droplet volume was fixed to 5 μL. For each sample, at least 15 independent droplets along the sample length were measured, with each droplet measured at least 10 times while relaxing. Drop shape analysis was carried out using Krüss ADVANCE software using a variety of drop profile fitting functions. Figure 1a,b shows sample images of pure water droplets on a superhydrophobic surface and on a silicone oil-infused surface. Contact angle hysteresis (CAH) measurements showed that our SLIPS demonstrated low CAH values, well below 1° for individual droplets. Each contact angle in the tables in the Supporting Information corresponds to a single SLIP surface, and the reported average and standard deviation correspond to 10–17 droplets along the central length of the SLIP surface giving error estimates of typically ±0.5° with a maximal value of ±1.9°.

### Surface Tension Measurements

All surface and interfacial tension measurements were conducted on the Krüss droplet shape analyzer (DSA25S) at room temperature (17–25 °C), using the pendant drop technique,^51−53^ where the profile of a drop suspended from a needle is fitted to the Young–Laplace equation to determine the surface tension. In each case a droplet of the higher density liquid is suspended from a needle within the liquid of lower density resulting in an increased pressure inside the pendant drop as a result of the interfacial tension between inner and outer phases.^35,54,55^ The shape of the pendant drop is determined by the balance between the interfacial tension and gravity forces, which deform the drop into a pear shape. For accurate results, needle size should be such that the Worthington number, *W*_o_, a nondimensional number that scales the drop volume by the theoretical maximum drop volume that can be sustained for the system, is appropriately large;^35^ here we ensured *W*_o_ ≥ 1. The liquid–liquid interfacial tension γ_LL_ can be determined from the equation according to Bashforth and Adams:^51^
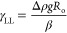
10where Δρ, *g*, *R*_o_, and β represent the density difference between the two liquids, the acceleration due to gravity, the drop radius of curvature at the apex, and a dimensionless shape factor parameter, respectively. In a typical pendant drop experiment the drop shape is numerically fitted to the Young–Laplace equation,^35^ which describes the pressure difference (i.e., Laplace pressure) between the areas inside and outside of a curved liquid surface/interface with the principal radii of curvature. From this fit the shape factor, and thus the interfacial tension, is determined from eq 10. The reliability of the method is extremely poor in the case of nearly spherical drops. In this case, any small change in the drop profile fit results in a large change in the measured surface tension.^56^

For liquid–liquid interfacial tension measurements we used the Krüss SC02 high-quality optical glass cuvette (36 × 36 × 30 mm). We used a wide range of metal and Teflon needles (adhesive dispensing) with different outer diameters depending on the working liquids. To avoid contamination one disposable plastic syringe (adhesive dispensing) was used for each sample. The pendant droplet volume was chosen slightly below the maximum volume at which the droplet would immediately detach from the needle. Each pendant droplet was left to relax for a time depending on the pair of working fluids, such that steady surface tension results could be collected and averaged. We were not able to measure the interfacial tension between water and some dark oils, such as avocado, due to their lack of transparency. For Krytox the method required a very long measurement time of several hours to reach equilibrium, even though evaporation was almost absent. Figure 1c,d provides examples of the pendant drop images in the case of water–air and water–silicone oil surface tension measurements.

## Results and Discussion

### Liquid–Liquid Interfacial Tensions from Apparent Contact Angles

To demonstrate the ability of the liquid Young’s law apparent contact angle on SLIPS method to determine the liquid–liquid interfacial tension for a wide range of liquid pairs, we used the following protocol: (1) Select several sets of infused-liquid and droplet liquids for our SLIPS ensuring the substrate solid is consistent with a stable SLIP surface. (2) Measure the apparent contact angles for the different liquid pairs. (3) Use the established pendant drop technique to measure the droplet liquid–vapor and infused-liquid–vapor surface tensions, and the infused-liquid-droplet liquid interfacial tension. (4) Use eq 8 to predict the lubricant–droplet interfacial tensions from the measured contact angles and droplet liquid–vapor, and infused-liquid–vapor surface tensions, thus testing both the cloaked and noncloaked cases. (5) Use the infused-liquid–droplet liquid interfacial tensions predicted in step 4 to calculate the spreading coefficients (eq 9) predicting whether noncloaking and cloaking occurs, and select the case which gives a sign consistent with the spreading criterion. (6) Compare the final infused-liquid–droplet liquid interfacial tensions predicted by our method to the ones directly measured using the pendant drop technique.

**Figure 2:**
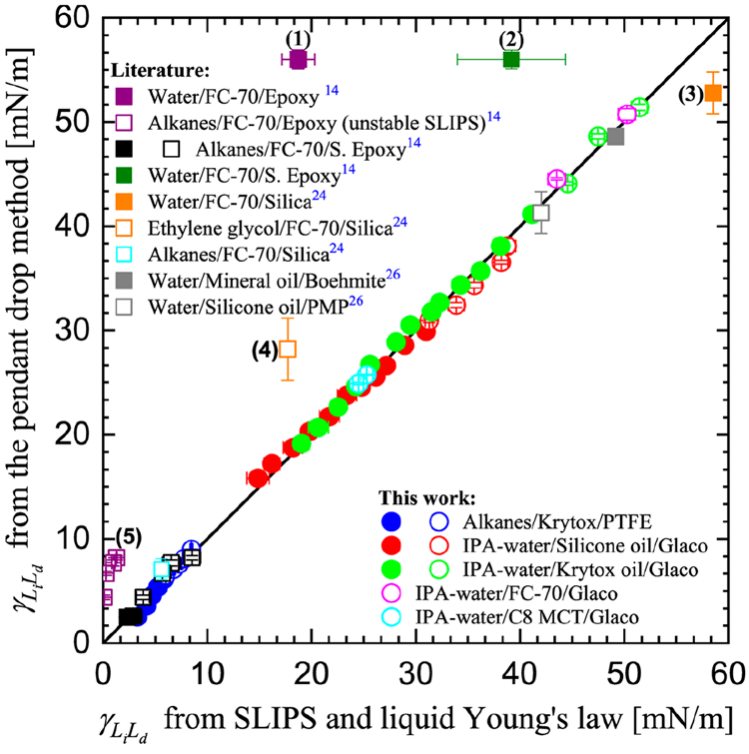
Comparison between liquid–liquid interfacial tension measured by the pendant drop method (*y*-axis) and those predicted by the new SLIPS method (*x*-axis). Results are reported for different pairs of liquids studied in this paper and reported in several literature studies.^14,24,27^ Solid and empty symbols refer, respectively, to noncloaked and cloaked droplets. For literature data we used contact angles and pendant drop interfacial tensions reported in those studies.

In Figure 2 we report the liquid–liquid interfacial tension results predicted by the new SLIPS method (*x*-axis) and compare them to the results measured directly by the pendant drop method (*y*-axis) (Tables S1–S3). Ideally, if the two methods perfectly agree with each other, all the data points should be located on the identity (or 45°) black line. Figure 2 includes our results (empty and solid circles) in addition to results summarized from the published literature (empty and solid squares).^14,24,27^ In Figure 2 empty and solid symbols are used to indicate whether the infused-liquid does or does not cloak the droplet. This distinction was made based on testing these two cloaking scenarios using eq 9 reported in the theory section. Since the figure includes a wide range of droplet and lubricating liquids we use a unique color for each droplet/lubricant/surface combination for both the cloaked and noncloaked cases.

As shown by the blue circles in Figure 2, we tested droplets of ten alkanes on Krytox-infused Teflon AF surfaces. While droplets of alkanes with low alkane-vapor surface tensions from 17.2 to 22.4 mN/m (i.e., pentane, hexane, heptane, octane, and nonane) were found to be noncloaked, alkanes with higher alkane–vapor surface tensions from 23.6 to 27.2 mN/m (i.e., decane, undecane, dodecane, tridecane, and hexadecane) were preferentially cloaked by Krytox. Our Krytox–alkanes interfacial tension results predicted by the new SLIPS method agree very well with the pendant drop results. Similar agreement was obtained for alkanes droplets on FC-70-lubricated S epoxy surfaces (black squares) reported by Wong et al.^14^

We also evaluated IPA–water droplets on Glaco superhydrophobic surfaces lubricated with silicone oil (red circles) and Krytox (green circles). Tested IPA–water droplets contained 0, 1, 2, 3, 4, 5, 6, 7, 8, 9, 10, 12, 14, 16, 18, and 20 vol % IPA. As can be seen from Figure 2, only droplets with large liquid–vapor surface tensions were found in the cloaked state for both silicone oil and Krytox lubricants, in agreement with what occurred in the case of alkanes on Krytox. For these two sets of samples we obtained very good agreement between liquid–liquid interfacial tensions predicted by the new SLIPS method and measured by the pendant drop method. Similar agreement was also obtained for water droplet containing 0 and 2 vol % IPA setting on the top of Glaco superhydrophobic surfaces lubricated with FC-70 (magenta circles) and C8MCT edible oil (cyan circles). In these two cases droplets were found to be in cloaked states, in agreement with the above results. From the full set of data across different types of infused-liquids and droplet liquids, the results reported showed excellent agreement between our new SLIPS method and pendant drop technique.

### Methodological Considerations for Measurements of Apparent Contact Angles

There are several data points from the literature included in Figure 2 (identified by labels 1–5, where 5 is the data points for an alkane series) which lie away from the line of agreement for the liquid–liquid interfacial tensions deduced from the apparent contact angle and pendant drop methods. In each case, we are able to identify the methodological issues that may explain these data points. Consider first the disagreement for the alkane droplets on FC-70-lubricated epoxy surfaces reported by Wong et al.^14^ (purple squares labeled 5). The data for this series of alkane droplets on SLIP surfaces can be explained as the authors’ reported observation that these SLIP surfaces become unstable due to the alkanes displacing the FC-70 infused-liquid lubricant. This suggests eq 7 was not satisfied for alkanes and FC-70 on these epoxy-based surfaces.

The next methodological consideration is whether or not the apparent contact angle measured was the tangent angle at the inflection point in the side profile of the droplet on a sufficiently thin layer of infused-liquid such that the wetting ridge is vanishingly small. If a finite height wetting ridge exists, the apparent contact angle measured as the tangent angle at the inflection point in the side profile image will be underestimated due to the wetting ridge rotation effect.^29^ The numerical effect of this type of methodological error in the measured apparent contact angle can be estimated for cloaked and noncloaked droplets (away from a cloaking transition) by considering θ_app_ = θ_app_^s^ ± Δθ, where the negative sign is chosen, and Δθ is the magnitude of the angular rotation; the positive sign would represent an overestimate. If this is substituted into eq 8 as the apparent contact angle and a series expansion performed to first order, it gives an underestimate (the negative sign) in the liquid–liquid interfacial tension of magnitude:
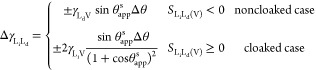
11We note that eq 11 does not take into account whether an under- or overestimate in the apparent contact angle in eq 8 alters the classification of a droplet as noncloaked or cloaked when using eq 9.

Equation 11 indicates that placing a baseline for the measurement of the apparent contact angle below the inflection point in the side profile of the droplet and measuring the tangent angle to the slope of the profile at the solid surface will result in an underestimate of the apparent contact angle and, hence, of the liquid–liquid interfacial tension. However, some reports in the literature have not used the tangent angle at the inflection point of the side profile of a droplet as the definition of the apparent contact angle, but have used the extrapolation of a circular arc profile or a fit to the Young–Laplace equation (ignoring the wetting ridge) from the apex of the droplet down to the solid surface (to within the thickness of the infused-liquid layer). If there is a visible wetting ridge, this approach may overestimate the apparent contact angle and, following a similar logic to eq 11, result in an overestimate compared to using a tangent angle at the inflection point in the slope of profile, although this inflection point itself would be an underestimate of θ_app_^s^ due to the wetting ridge rotation effect. A further possibility is that the placement of the baseline for measuring the inflection angle could be above its true location, and this would lead to an underestimate in the contact angle.

**Figure 3:**
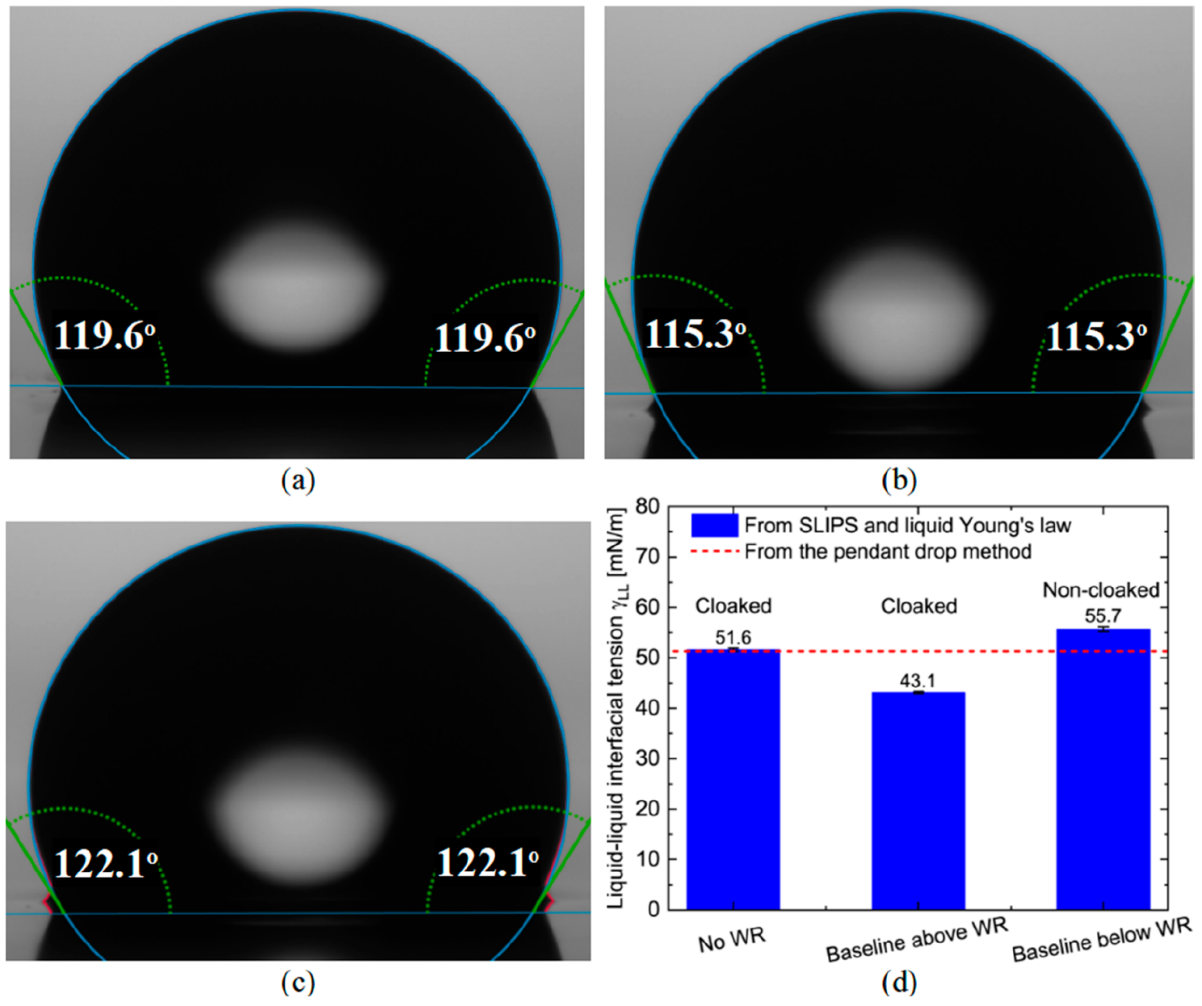
Wetting ridges (WR) and different baseline choices induce errors in the measured apparent contact angle and liquid–liquid interfacial tension predicted by our new SLIPS method. Results reported for a pure water droplet on Krytox-infused Glaco-based SLIPS. (a) Ideal droplet without WR. (b) Droplet with WR and baseline chosen above WR. (c) Droplet with WR and baseline chosen below WR. (d) Water–Krytox interfacial tension predicted by our new SLIPS method compared to the value measured by the pendant drop method.

To illustrate these ideas, Figure 3a compares a water droplet on top of a thin Krytox-infused Glaco-coated substrate with a water droplet on top of a Krytox-infused Glaco-coated substrate, but with excess Krytox (Figure 3b,c). In these figures the red profiles show the actual droplet shapes, and the blue profiles show the Young–Laplace fitting of these shapes using the algorithm provided with the Krüss DSA25 instrument. The fitting baselines are shown by the horizontal blue lines. For the profile in Figure 3a, there is no wetting ridge and therefore only one way to choose the fitting baseline, resulting in an accurate apparent contact angle of θ_app_^s^ = 119.6°. For the droplet on SLIPS with excess Krytox, the use of a baseline above the wetting ridge (Figure 3b) results in an underestimated apparent contact angle of θ_app_^s^ = 115.3°. In contrast, choosing a baseline below the wetting ridge (Figure 3c) results in an overestimated apparent contact angle of θ_app_^s^ = 122.1°. The trends in these under-/overestimates are consistent with the expectations below. Figure 3d shows the water–Krytox interfacial tensions predicted from the three values of contact angles in Figure 3a–c (blue columns). As a reference, we also show the water–Krytox interfacial tension as measured by the pendant drop method (red dashed line). Choosing the baseline below the wetting ridge not only predicts an overestimated water–Krytox interfacial tension, but also leads to a wrong characterization of the water droplet cloaking behavior.

We now return to considering those literature data in Figure 2 which are away from the line of agreement between the apparent contact angle and pendant drop methods for determining the liquid–liquid interfacial tension. In the case of water droplets on FC-70-lubricated silanized (black empty square in Figure 2 labeled 2) and nonsilanized (purple empty square in Figure 2 labeled 1) epoxy surfaces,^14^ Wong et al. reported water contact angles of 113.1° and 92.6° measured using the manufacturer-provided software based on a spherical cap droplet profile. Our measured contact angles of water on Glaco lubricated with FC-70 and Krytox were 119.1° ± 0.4° and 119.5° ± 0.2°, respectively. The similarity between these two contact angles is expected since FC-70 and Krytox have very similar liquid–vapor surface tensions (i.e., 17.35 ± 0.04 mN/m for FC-70 and 17.41 ± 0.02 mN/m for Krytox). Therefore, the water contact angles reported by Wong et al.^14^ appear to be underestimated, which would result in underestimates for the water–FC-70 interfacial tension. One possibility here is that this could be caused by placing a baseline above the inflection point in the droplet profile to ensure the portion of the droplet shape used was a spherical cap undisturbed by a wetting ridge.

In the cases of water–FC-70 (orange filled square in Figure 2 labeled 3) and ethylene glycol-FC-70 (orange empty square in Figure 2 labeled 4), the obtained liquid–liquid interfacial tension predictions are based on contact angles reported by Schellenberger et al.,^24^ who used a Young–Laplace fitting method on droplets with visible wetting ridges. In both cases they appear to have chosen the droplet baseline below the wetting ridges (see droplet profiles in Figure 3 in Schellenberger et al.^24^). This suggests that the deduced interfacial tensions for these three liquid–liquid combinations in Figure 2 based on the Schellenberger et al.^24^ reported apparent contact angles are likely to be overestimates. This would be consistent with attributing an overestimate of the liquid–liquid interfacial tension for water–FC-70 (orange filled square labeled 3) in our Figure 2 to the baseline placement and Young–Laplace contact angle measurement method they used. However, it would not explain the underestimate ethylene glycol-FC-70 (orange empty square in Figure 2 labeled 4). To obtain an underestimate in the liquid–liquid interfacial tension deduced from the contact angle, the wetting ridge would need to be sufficiently large that the underestimate from the wetting ridge rotation effect^29^ would outweigh the overestimate from the baseline placement and Young–Laplace contact angle measurement method they used. Indeed, the wetting ridges visible in the droplet profiles in Figure 3 in Schellenberger et al.^24^ are visibly higher up on the profile for the droplets of ethylene glycol compared to the droplets of water.

### Methodological Considerations for the Pendant Drop Method

**Figure 4:**
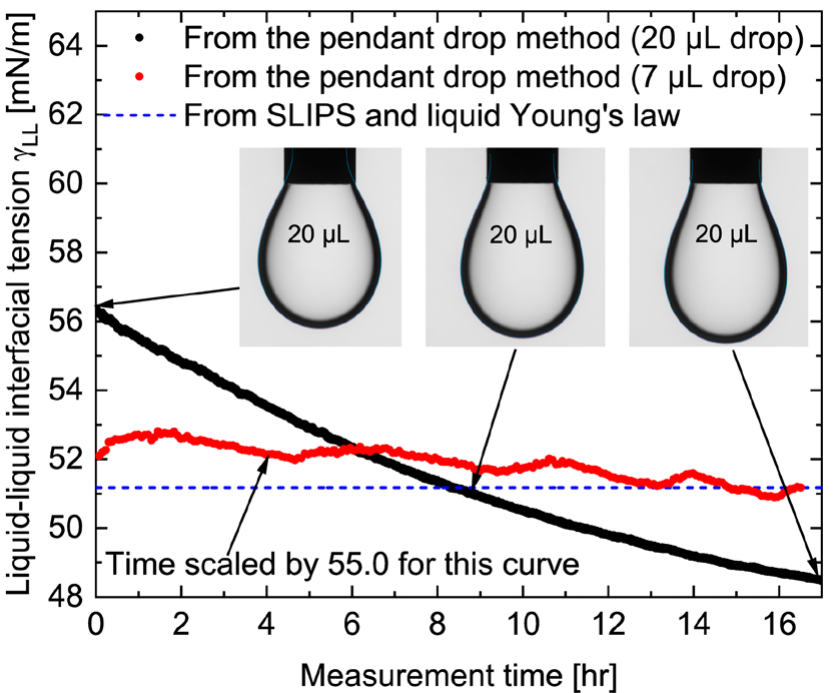
Unreliability of the pendant drop method in measuring the interfacial tension between pure water and Krytox. When we used large pendant droplets (black circles) the water–Krytox interfacial tension could not reach equilibrium after long measurement time and continued to decrease to nonphysical values. When we used a small droplet size of 7.0 μL (red circles) we could obtain a converging result in a few minutes, which agrees very well with the interfacial tension predicted by our SLIPS method (blue dashed line).

We also found that reliably obtaining accurate water–FC-70 and water–Krytox interfacial tensions using the pendant drop method required particular care to ensure the pendant droplet shape was in equilibrium. Best practice guidance suggests using the largest possible pendant drop volumes for a given needle size and collecting data for long periods in an attempt to reach equilibrium. Figure 4 shows one example of one of our failed pendant drop experiments for the water–Krytox combination. The liquid–liquid interfacial tension starts with a large value, similar to the one reported by Wong et al.,^14^ and then continues to decrease but never reaches equilibrium, even after very long measurement times. From the pendant drop snapshots (insets in Figure 4) it appears that when the drop volume is large the drop shape continues to be deformed by gravity, and the shape parameter β does not reach equilibrium. Since the density of Krytox is approximately double the density of water, the drop deformation is dominated actually by gravity for large droplets. We found that smaller pendant drop volumes of around 7.0 μL (red circles in Figure 4) gave a reasonable balance between the gravitational and interfacial forces, such that a stable shape parameter could be obtained in a reasonable equilibration time. Although limitations of the pendant drop method were thoroughly discussed in the literature,^35,56,57^ the failure of the method in measuring interfacial tension between two particular liquids can be very complex to understand since it can be caused by a combination of factors.

In Figure 4 the disagreement between the liquid–liquid interfacial tensions predicted by the SLIPS method and those measured by the pendant drop method, despite a large *W*_o_ number, may in some cases be due to the failure of the pendant drop technique to correctly handle certain liquid combinations: for example, when there are large density differences, when one liquid is strongly polar, or when there is a migration of surface active components from the bulk to the surface. Our SLIPS method has predicted interfacial tensions of 50.3 ± 0.8 and 51.2 ± 0.5 mN/m for the water–FC-70 and water–Krytox combinations, respectively. The fact that these two values are very close to each other, within experimental uncertainty, is expected since FC-70 and Krytox have very similar liquid–vapor surface tensions. Furthermore, the predicted water–Krytox interfacial tension agrees well with the general trend of IPA–water–Krytox interfacial tension as a function of the IPA content (see the Supporting Information). Wong et al. have reported water–FC-70 interfacial tension of 56.0 ± 0.9 mN/m,^14^ larger than the value 53.0 ± 2.0 mN/m reported by Schellenberger et al.^24^ While Wong et al. did not report the measurement details, Schellenberger et al.^24^ did not report the time variation of the measured water–FC-70 interfacial tension, along with the time variation of interfacial tensions of other liquid combinations reported in the same study.

### Zisman Method on Liquid Surfaces

The Zisman method for assessing the wettability of a solid surface through measurement of contact angles for a sequence of liquids, and from them a deduction of a critical surface tension (CST) for the solid, is a significant concept in the wetting literature.^43−45^ Since the liquid Young’s law defines clearly an apparent contact angle on a thin liquid surface, it is our hypothesis that the Zisman method should also be applicable to SLIP surfaces (i.e., the infusing-liquid surface is analogous to the solid surface). We anticipate that the liquid–vapor surface tension may need to be reinterpreted as an effective surface tension to take into account the effect of cloaking of droplets by some infused-liquids. Interestingly, since a droplet of the infused-liquid itself will spread on a SLIP surface, we expect the value of the critical surface tension extrapolated from a sequence of measurements of droplets with different liquids should either give the same value as the infused-liquid–vapor interfacial tension or be less than it, i.e., γ_C_ ≤ γ_L_i_V_. To study the dependence of CST on the lubricant type we investigated the following two series of liquids and SLIPS: (1) 10 alkanes forming droplets on Krytox-infused Teflon AF surfaces (to avoid the alkanes displacing the Krytox) (Table S1), (2) a series of IPA–water solutions forming droplets on Krytox-infused Glaco surfaces (Table S2), and (3) a series of IPA–water solutions forming droplets on silicone oil-infused Glaco surfaces (Table S3). The apparent contact angle ranges for these three systems are 34.7–69.8°, 93.0–119.6°, and 80.2–108.3°, respectively, with contact angle hysteresis typically ∼±0.5° and in all cases less than ±2°. The liquid–vapor surface tensions of Krytox and silicone oil are γ_L_i_V_^Krytox^ = (17.41 ± 0.02) mN/m and γ_L_i_V_^silicone-oil^ = (20.2 ± 0.1) mN/m, respectively, and hence our hypothesis is that γ_C_^Krytox^ ≤ 17.41 and γ_C_^silicone-oil^ ≤ 20.2 mN/m, respectively. All of our data together with data from the literature are given in the Supporting Information (Tables S1–S4).

### Original Zisman Plot

**Figure 5:**
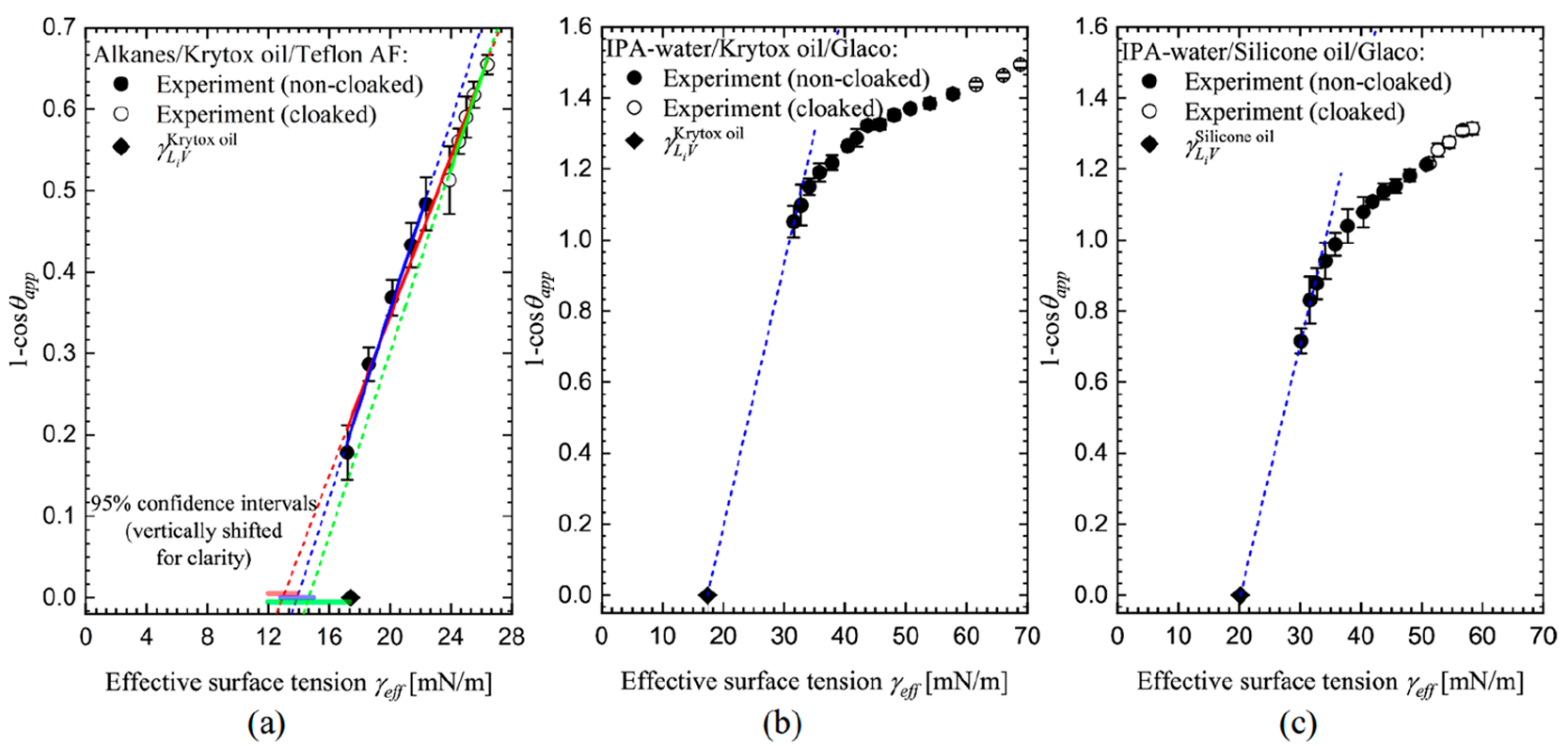
Zisman plots of data for (a) alkane droplets on Krytox-infused Teflon AF, (b) IPA–water droplets on Krytox-infused Glaco, and (c) IPA–water droplets on silicone oil-infused Glaco, following the original Zisman method.^43−45^ Solid and empty symbols indicate noncloaked and cloaked droplets, respectively. Effective surface tension in the case of noncloaked droplets corresponds to the liquid–vapor surface tension. Linear fitting in panel a was performed on either all (red lines), noncloaked (blue lines), or cloaked (green lines) data points; solid lines present fitting results in the employed data ranges, and dashed lines represent fit extrapolations. The estimated critical surface tensions are summarized in Table 1. Semitransparent horizontal bars indicate the lower and upper limits of the 95% confidence interval of estimated critical surface tensions. Dashed lines in panels b and c are guides to the eye joining the infused-liquid surface tension to the data points with the data for the droplets with the lowest values of surface tension.

We first consider data for the dependence of (1 – cos θ_app_) on the effective liquid–vapor surface tension γ_eff_ for each droplet (Figure 5). In each case, we indicate whether the measured apparent contact angle implies through eq 9 that the droplet is noncloaked, i.e., γ_eff_ = γ_L_i_V_ (filled symbols), or cloaked, i.e., γ_eff_ = γ_L_d_L_i__ + γ_L_i_V_ (empty symbols), and also indicate the lubricant–vapor interfacial tension (solid diamond symbols). We observe that the data shows droplets transition from cloaked to noncloaked as the effective surface tension reduces in value in Figure 5. Motivated by our hypothesis that γ_C_ ≤ γ_L_i_V_, we define a Δγ by the equation
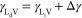
12and consider the spreading coefficient for the infused-liquid on the droplet liquid
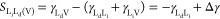
13

Thus, droplets will be noncloaked for Δγ < γ_L_d_L_i__ and cloaked for Δγ ≥ γ_L_d_L_i__ with a transition in the trend between the two occurring when Δγ < γ_L_d_L_i__, i.e.
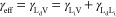
14

Figures 5a–5c each show three types of linear fits for the Krytox-infused Teflon AF, silicone oil-infused Glaco, and Krytox-infused Glaco substrates. The red solid line in Figure 5a uses all data points for the alkane series of droplets on Krytox-infused-Teflon AF substrate, and the dashed red line extrapolates this to predict γ_C_^Krytox^ = (13.0 ± 0.9) mN/m. Fitting the cloaked and noncloaked data points separately gives predictions of γ_C_^Krytox^ = (14.7 ± 2.7) mN/m and γ_C_^Krytox^ = (13.9 ± 1.1) mN/m, respectively. All three predictions are lower than the measured value of the Krytox liquid–vapor interfacial tension of γ_L_i_V_^Krytox^ = (17.41 ± 0.02) mN/m. Moreover, pentane (C_5_H_12_) which has a surface tension of γ_L_d_V_^pentane^ = 17.2 mN/m (i.e., below that of Krytox), forms a partially wetting droplet with an apparent contact angle of 34.7° ± 1.7°. This is consistent with the hypothesis that γ_C_ ≤ γ_L_i_V_. The estimated CST also appears to be consistent with the expectation that the wetting properties of a surface depend on both the chemical structure (e.g., −CF_2_ and −CF_3_ end groups) and the physical state of the surface^58^ (see Table 10-10, Adamson and Gast^1^ and Schindere and Houser^49^). Thus, for liquid surfaces whether the surface is in the form of an infused-liquid within a SLIPS or bulk liquid may be an analogy to whether the physical state of a solid is a crystal, monolayer, or polymer.

In Figure 5b,c it is visually obvious that extrapolation of data for the IPA–water solution droplets is difficult because the droplet apparent contact angles do not sufficiently approach 0° for a linear fit extrapolation at lower effective surface tensions to be performed with accuracy. We have therefore provided blue dashed lines in Figure 5b,c joining the value of the infused-liquid–vapor surface tensions (solid diamond symbols using γ_L_i_V_^Krytox^ = (17.41 ± 0.02) mN/m and γ_L_i_V_^silicone-oil^ = (20.2 ± 0.1) mN/m, with the data points for the lowest surface tensions of the IPA–water solution droplets on Krytox-infused Glaco and silocone oil-infused Glaco substrates. While these dashed lines are only guides to the eye and not predictive fits, they suggest data might be consistent with the hypothesis that the critical surface tensions are no larger than the values of the infused-liquid–vapor surface tensions.

### Modified Zisman Plot

**Figure 6:**
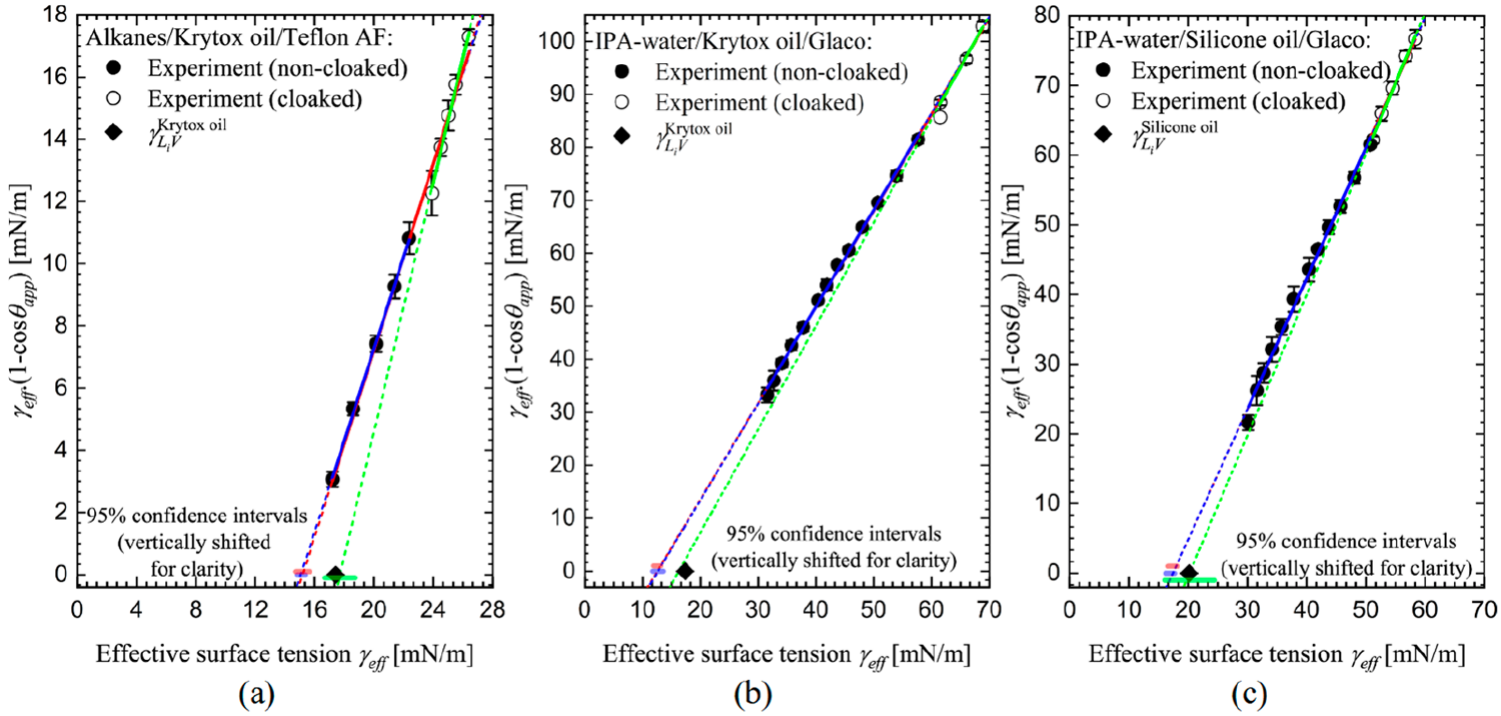
Modified Zisman plots of data for (a) alkane droplets on Krytox-infused Teflon AF, (b) IPA–water droplets on Krytox-infused Glaco, and (c) IPA–water droplets on silicone oil-infused Glaco.^41,51^ Solid and empty symbols refer to noncloaked and cloaked droplets, respectively. Effective surface tension in the case of noncloaked droplets corresponds to the liquid–vapor surface tension. Linear fitting was performed on either full (red lines), noncloaked (blue lines), or cloaked (green lines) data points. Solid lines present fitting results in the employed data ranges, and dashed lines represent fit extrapolations. The estimated critical surface tensions are summarized in Table 1. Semitransparent horizontal bars indicate the lower and upper limits of the 95% confidence interval of estimated critical surface tensions.

Neumann et al.^45,59^ suggested that the Zisman method should be modified by plotting γ_LV_ cos θ [or equivalently γ_LV_(1 – cos θ)] against the liquid–vapor surface tension γ_LV_. Taking into account the possibility of cloaking or noncloaking on SLIP surfaces, Figure 6 therefore shows our data plotted as γ_eff_(1 – cos θ_app_) against the effective liquid–vapor surface tension γ_eff_. The superior quality of the linear fitting is evident in Figure 6 compared to Figure 5, particularly in the case of SLIPS based on silicone oil. Fitting to the data for noncloaked droplets, Figure 6a,b gives predictions of γ_C_^Krytox^ = (15.1 ± 0.3) mN/m and γ_C_^Krytox^ = (12.7 ± 0.6) mN/m, respectively, compared to the measured value of the Krytox liquid–vapor interfacial tension γ_L_i_V_^Krytox^ = (17.41 ± 0.02) mN/m. Fitting to the data for noncloaked droplets on silicone oil-infused Glaco surfaces predicts γ_C_^silicone-oil^ = (17.4 ± 1.1) mN/m compared to γ_L_i_V_^silicone-oil^ = (20.2 ± 0.1) mN/m. All three cases appear consistent with the hypothesis that γ_C_ ≤ γ_L_i_V_ and further suggest that the CST is less than the infused-liquid vapor surface tension. The higher values of CST on these silicone oil-infused SLIPS involving CH_3_ end groups compared with the perfluoroalkylether-based Krytox-infused SLIPS involving CF_2_CF_3_ end groups are consistent with expectations with solid surfaces (for comparisons of CST for solid surfaces see Table 10-10 in Adamson and Gast^1^ and Schindere and Houser^49^).

**Table 1:** Summary of Critical Surface Tensions Using the Original and Modified Zisman Plots

solid substrate	infused-liquid, L_i_	droplet liquid, L_d_	γ_L_i_V_ [mN/m]	fitted data range	γ_C_^*i*^ [mN/m]	Zisman method
Teflon AF	Krytox	alkanes	17.41 ± 0.02	all	13.0 ± 0.9	original
				noncloaked	13.9 ± 1.1	original
				cloaked	14.7 ± 2.7	original
Teflon AF	Krytox	alkanes	17.41 ± 0.02	all	15.2 ± 0.5	modified
				noncloaked	15.1 ± 0.3	modified
				cloaked	17.7 ± 1.0	modified
Glaco	Krytox	IPA–water	17.41 ± 0.02	all	12.6 ± 0.6	modified
				noncloaked	12.7 ± 0.6	modified
				cloaked	16.3 ± 3.9	modified
Glaco	silicone oil	IPA–water	20.2 ± 0.1	all	17.4 ± 0.6	modified
				noncloaked	17.4 ± 1.1	modified
				cloaked	20.2 ± 2.2	modified

## Conclusions

In this paper, we have considered how a liquid analogue of Young’s law allows the concept of the wettability of liquid surfaces on slippery liquid-infused porous solid surfaces (SLIPS) to be developed in analogy to the wettability of a solid surface. Motivated by contact angle measurements of droplets on solid surfaces, we have shown how apparent contact angles on SLIPS can be used as a new practical method to measure liquid–liquid interfacial tensions. This has emphasized the importance of the definition of the apparent contact angle as the tangent angle measured at the inflection point in the droplet profile and the validity of the liquid Young’s law as the limiting case of a vanishingly small wetting ridge. We have also shown that droplets of a homologous series of alkanes can be presented using a Zisman plot and a critical surface tension (CST) for the liquid surface in the SLIPS deduced by a linear extrapolation of the data. Our data further suggests that droplets of water–isopropyl-alcohol (IPA) solutions on SLIPS can be presented using a modified Zisman plot and critical surface tensions deduced. In all the cases we studied, the deduced critical surface tensions were less than the infused-liquid–vapor surface tensions consistent with the hypothesis that the upper bound on the CST is the infused-liquid–vapor surface tension.

## Supporting Information Available

Apparent contact angles used to estimate liquid–liquid interfacial tensions and pendant drop deduced liquid–vapor and interfacial tensions; apparent contact angles and liquid–vapor and liquid–liquid interfacial tensions for droplets of alkanes on Krytox-infused Teflon AF, droplets of IPA/water solutions on Krytox-infused Glaco, and droplets of IPA/water solutions on silicone oil-infused Glaco; and a summary of liquid–liquid interfacial tensions deduced from literature reports (PDF)
