# Supplementary material for: The Liquid Young’s Law on SLIPS: Liquid–Liquid Interfacial Tensions and Zisman Plots
Source: Langmuir. 2022 Aug 3;38(32):10032–42. doi: 10.1021/acs.langmuir.2c01470 (PMC9387105; doi:10.1021/acs.langmuir.2c01470)
Supplement: Supplementary file 1 — la2c01470_si_001.pdf [file la2c01470_si_001.pdf]

Supporting Information

The Liquid Young's Law on SLIPS: Liquid-Liquid Interfacial Tensions and Zisman Plots

Glen McHale*, Nasser Afify$, Steven Armstrong, Gary G. Wells and Rodrigo Ledesma-Aguilar

Institute for Multiscale Thermofluids, School of Engineering, The University of Edinburgh, Edinburgh EH9 3FB, UK.

*Email: glen.mchale@ed.ac.uk

$Email: n.afify@ed.ac.uk

Table S1. Apparent contact angle and surface tension results for the alkane droplet series on Krytox-infused Teflon AF substrate with $\gamma_{L_iV} = 17.41 \pm 0.02$ mN/m. The droplet liquid type, apparent contact angle, droplet liquid-vapour surface tension, infused-liquid-vapour surface tension, spreading coefficient, and liquid-liquid interfacial tension between the droplet liquid and the infused-liquid are given by L_d, θ_{app}, γ_{L_dV}, $S_{L_iL_d(V)}$ and $\gamma_{L_dL_i}$, respectively. $S_{L_iL_d(V)}$ shows two possible cases according to eq 8 and indicates the case consistent with conditions from eq 9. Contact angle and surface tension measurements were carried at temperatures between 19-21°C.

L_d	θ_{app} [°]	γ_{L_dV} [mN/m]	$S_{L_iL_d(V)}$ [mN/m]		Cloaked (Y/N)	$\gamma_{L_dL_i}$ [mN/m] SLIPS Contact Angle Method	$\gamma_{L_dL_i}$ [mN/m] Pendant Drop Method
			Non-cloaked	Cloaked			
Pentane (C_5H_{12})	34.7±1.7	17.20	-3.49	~~-1.91~~	N	3.28±0.32	2.52±0.03
Hexane (C_6H_{14})	44.5±0.9	18.60	-2.95	~~-1.72~~	N	4.14±0.22	3.57±0.04
Heptane (C_7H_{16})	50.8±0.8	20.14	-1.96	~~-1.20~~	N	4.69±0.24	4.58±0.01
Octane (C_8H_{18})	55.5±1.0	21.40	-1.29	~~-0.82~~	N	5.28±0.32	5.38±0.01
Nonane (C_9H_{20})	58.9±1.1	22.37	-0.90	~~-0.59~~	N	5.86±0.40	6.13±0.04
Decane ($C_{10}H_{22}$)	60.9±1.4	23.60	~~0.27~~	0.18	Y	6.01±0.34	6.50±0.05
Undecane ($C_{11}H_{24}$)	63.9±0.5	24.66	~~0.68~~	0.47	Y	6.78±0.14	7.10±0.01
Dodecane ($C_{12}H_{26}$)	65.8±0.8	25.35	~~0.92~~	0.65	Y	7.29±0.23	7.60±0.01
Tridecane ($C_{13}H_{28}$)	67.5±0.5	25.90	~~0.98~~	0.71	Y	7.78±0.16	8.10±0.01
Hexadecane ($C_{16}H_{34}$)	69.8±0.4	27.20	~~1.76~~	1.31	Y	8.48±0.13	8.99±0.01

Table S2. Apparent contact angle and surface tension results for the IPA-water droplet series on Krytox-infused Glaco substrate with $\gamma_{L_iV} = 17.41 \pm 0.02$ mN/m. The %IPA for the droplet, apparent contact angle, droplet liquid-vapour surface tension, infused-liquid-vapour surface tension, spreading coefficient, and liquid-liquid interfacial tension between the droplet liquid and the infused-liquid are given by L_d %IPA, θ_{app}, γ_{L_dV}, $S_{L_iL_d(V)}$ and $\gamma_{L_dL_i}$, respectively. $S_{L_iL_d(V)}$ shows two possible cases according to eq 8 and indicates the case consistent with conditions from eq 9. The droplet liquid-vapour surface tension data were taken from Park *et al.*[S1] Contact angle and surface tension measurements were carried at temperatures between 19-21°C.

L_d %IPA vol/vol	θ_{app} [°]	γ_{L_dV} [mN/m]	$S_{L_iL_d(V)}$ [mN/m]		Cloaked (Y/N)	$\gamma_{L_dL_i}$ [mN/m] SLIPS Contact Angle Method	$\gamma_{L_dL_i}$ [mN/m] Pendant Drop Method
			Non-cloaked	Cloaked			
0	119.6±0.3	72.71	~~1.94~~	3.84	Y	51.46±0.78	51.41±0.30
1	117.6±0.3	67.33	~~1.31~~	2.44	Y	47.49±0.61	48.61±0.26
2	116.0±0.2	62.15	~~0.10~~	0.17	Y	44.57±0.48	44.10±0.15
3	114.3±0.4	57.71	-0.87	~~-1.48~~	N	41.17±0.43	41.16±0.11
4	112.6±0.5	53.94	-1.60	~~-2.60~~	N	38.13±0.46	38.10±0.37
5	111.7±0.3	50.75	-2.86	~~-4.54~~	N	36.20±0.26	35.69±0.08
6	110.6±0.4	48.04	-3.67	~~-5.66~~	N	34.30±0.35	34.36±0.13
7	109.0±0.4	45.71	-4.00	~~-5.94~~	N	32.30±0.38	32.68±0.22
8	108.8±0.4	43.69	-5.24	~~-7.74~~	N	31.52±0.32	31.79±0.24
9	106.7±0.7	41.94	-4.95	~~-6.95~~	N	29.48±0.56	30.53±0.22
10	105.4±0.3	40.40	-5.12	~~-6.97~~	N	28.11±0.25	28.88±0.11
12	102.5±0.6	37.83	-5.20	~~-6.64~~	N	25.63±0.43	26.72±0.05
14	101.0±0.8	35.79	-5.85	~~-7.23~~	N	24.23±0.50	24.62±0.08
16	98.7±0.7	34.13	-5.83	~~-6.86~~	N	22.54±0.43	22.66±0.09
18	95.6±1.6	32.75	-5.29	~~-5.87~~	N	20.63±0.96	20.70±0.07
20	93.0±1.3	31.60	-4.86	~~-5.13~~	N	19.05±0.73	19.16±0.10

Table S3. Apparent contact angle and surface tension results for the IPA-water droplet series on silicone oil-infused Glaco substrate with $\gamma_{L_iV} = 20.22 \pm 0.05$ mN/m. The %IPA for the droplet, apparent contact angle, droplet liquid-vapour surface tension, infused-liquid-vapour surface tension, spreading coefficient, and liquid-liquid interfacial tension between the droplet liquid and the infused-liquid are given by L_d %IPA, θ_{app}, γ_{L_dV}, $S_{L_iL_d(V)}$ and $\gamma_{L_dL_i}$, respectively. $S_{L_iL_d(V)}$ shows two possible cases according to eq 8 and indicates the case consistent with conditions from eq 9. The droplet liquid-vapour surface tension data were taken from Park *et al.*[S1] Contact angle and surface tension measurements were carried at temperatures between 19-21°C.

L_d %IPA vol/vol	θ_{app} [°]	γ_{L_dV} [mN/m]	$S_{L_iL_d(V)}$ [mN/m]		Cloaked (Y/N)	$\gamma_{L_dL_i}$ [mN/m] SLIPS Contact Angle Method	$\gamma_{L_dL_i}$ [mN/m] Pendant Drop Method
			Non-cloaked	Cloaked			
0	108.3±0.5	72.71	~~9.41~~	13.73	Y	38.76±0.82	38.10±0.70
1	107.9±0.4	67.33	~~6.17~~	8.92	Y	38.20±0.63	36.54±0.17
2	106.0±0.5	62.15	~~4.59~~	6.34	Y	35.60±0.70	34.30±0.27
3	104.6±0.6	57.71	~~2.69~~	3.60	Y	33.89±0.78	32.44±0.23
4	102.4±0.3	53.94	~~1.92~~	2.44	Y	31.28±0.37	30.96±0.20
5	102.3±0.2	50.75	-0.45	~~-0.57~~	N	30.98±0.27	29.89±0.17
6	100.5±0.5	48.04	-1.11	~~-1.36~~	N	28.94±0.44	28.58±0.17
7	98.8±0.6	45.71	-1.68	~~-1.99~~	N	27.18±0.51	26.63±0.13
8	97.8±0.7	43.69	-2.69	~~-3.12~~	N	26.17±0.56	25.55±0.09
9	96.3±0.3	41.94	-3.06	~~-3.43~~	N	24.78±0.27	24.57±0.09
10	94.5±1.2	40.40	-3.22	~~-3.49~~	N	23.40±0.91	23.79±0.13
12	92.3±0.4	37.83	-4.10	~~-4.26~~	N	21.71±0.96	21.75±0.15
14	89.3±0.9	35.79	-4.21	~~-4.16~~	N	19.78±0.61	20.29±0.17
16	86.6±1.5	34.13	-4.29	~~-4.05~~	N	18.20±0.92	18.75±0.12
18	83.0±1.3	32.75	-3.67	~~-3.27~~	N	16.20±0.76	17.23±0.10
20	80.2±1.9	31.60	-3.47	~~-2.97~~	N	14.85±1.08	15.82±0.19

Table S4. Summary of literature data used in Figure 2. The droplet liquid, infusing liquid, apparent contact angle, droplet liquid-vapour surface tension, infused-liquid-vapour surface tension, spreading coefficient, and liquid-liquid interfacial tension between the droplet liquid and the infused-liquid are given by L_d, L_d, θ_{app}, γ_{L_dV}, $S_{L_iL_d(V)}$ and $\gamma_{L_dL_i}$, respectively. The $S_{L_iL_d(V)}$ and $\gamma_{L_iL_d}$ calculations, and the cloaking decisions were produced using eq 8 and eq 9.

Solid surface	L_d	L_i	θ_{app} [°]	γ_{L_dV} [mN/m]	γ_{L_iV} [mN/m]	$S_{L_iL_d(V)}$ [mN/m]	Cloaked (Y/N)	$\gamma_{L_iL_d}$ [mN/m] SLIPS Contact Angle Method	$\gamma_{L_iL_d}$ [mN/m] Pendant Drop Method	Ref.
S. Epoxy	Water	FC-70	113.1±2.8	72.4±0.1	17.1±0.3	16.12	Y	39.18±5.21	56.0±0.9	[S2]
	Hexadecane ($C_{16}H_{34}$)		70.5±2.0	27.2±0.2		1.56	Y	8.54±0.81	8.2±0.2	
	Tridecane ($C_{13}H_{28}$)		63.5±2.8	25.9±0.1		2.25	Y	6.55±0.87	7.7±0.3	
	Decane ($C_{10}H_{22}$)		60.0±2.8	23.6±0.1		0.80	Y	5.70±0.78	6.7±0.2	
	Octane (C_8H_{18})		50.7±3.0	21.4±0.2		0.46	Y	3.84±0.62	4.4±0.2	
	Hexane (C_6H_{14})		40.1±4.2	18.6±0.5		-1.37	N	2.87±0.86	2.6±0.1	
	Pentane (C_5H_{12})		30.8±3.1	17.2±0.5		-2.23	N	2.33±0.38	2.5±0.1	
Epoxy	Water		92.6±1.8	27.2±0.2		36.57	Y	18.73±1.57	56.0±0.9	
	Hexadecane ($C_{16}H_{34}$)		30.6±0.4	25.9±0.1		8.82	Y	1.28±0.06	8.2±0.2	
	Tridecane ($C_{13}H_{28}$)		26.9±1.7	23.6±0.1		7.82	Y	0.98±0.15	7.7±0.3	
	Decane ($C_{10}H_{22}$)		14.2±0.7	21.4±0.2		6.23	Y	0.27±0.03	6.7±0.2	
	Octane (C_8H_{18})		7.9±0.7	27.2±0.2		4.22	Y	0.08±0.02	4.4±0.2	
Silica	Water		124.3	72.1	17.9	-4.33	N	58.50	52.8±2.0	[S3]
	ethylene glycol		89.6	47.3		11.75	Y	17.70	28.2±3.0	
	Hexadecane ($C_{16}H_{34}$)		58.5	28.0		4.49	Y	5.60	7.1±1.0	
PMP	Water	Silicone oil	110.0	72.4	20.6	9.78	Y	42.0	41.3	[S4]
Boehmite	Water	Mineral oil	105.4	72.4	30.0	-6.83	N	49.2	48.6	

REFERENCES

(S1) Park, J-G; Lee, S-H; Ryu, J-S; Hong, Y-K; Kim, T-G; Busnaina, A.A. Interfacial and Electrokinetic Characterization of IPA Solutions Related to Semiconductor Wafer Drying and Cleaning. *J. Electrochem. Soc.* **2006**, *153* (9), G811-G814.

(S2) Wong, T.-S.; Kang, S. H.; Tang, S. K. Y. Y.; Smythe, E. J.; Hatton, B. D.; Grinthal, A.; Aizenberg, J. Bioinspired Self-Repairing Slippery Surfaces with Pressure-Stable Omniphobicity. *Nature* **2011**, *477* (7365), 443–447.

(S3) Schellenberger, F.; Xie, J.; Encinas, N.; Hardy, A.; Klapper, M.; Papadopoulos, P.; Butt, H.; Vollmer, D. Direct Observation of Drops on Slippery Lubricant-Infused Surfaces. *Soft Matter* **2015**, *11* (38), 7617–7626.

(S4) Kreder, M. J.; Daniel, D.; Tetreault, A.; Cao, Z.; Lemaire, B.; Timonen, J. V. I.; Aizenberg, J. Film Dynamics and Lubricant Depletion by Droplets Moving on Lubricated Surfaces. *Phys. Rev. X* **2018**, *8* (3), 31053.

(S5) Jasper, J. J. The Surface Tension of Pure Liquid Compounds. J. Phys. Chem. Ref. Data **1972**, *1* (4), 841-1010.

(S6) Piñeiro, M. M.; García, J.; de Cominges, B. E.; Vijande, J.; Valencia, J. L.; Legido, J. L. Density and surface tension variation with temperature for n-nonane+1-hexanol. Fluid Phase Equilib. **2006**, *245* (1), 32-36.
